# Morphometric Analysis of the Common Raccoon Dog (*Nyctereutes procyonoides*) Teeth in Lithuania

**DOI:** 10.3390/vetsci12040338

**Published:** 2025-04-05

**Authors:** Eugenijus Jurgelėnas, Sigita Kerzienė, Linas Daugnora, Daniel Makowiecki

**Affiliations:** 1 Department of Anatomy and Physiology, Veterinary Academy, Lithuanian University of Health Sciences, Tilžės Str. 18, LT-47181 Kaunas, Lithuania; 2 Department of Animal Breeding, Veterinary Academy, Lithuanian University of Health Sciences, Tilžės Str. 18, LT-47181 Kaunas, Lithuania; sigita.kerziene@lsmu.lt; 3 Institute of Baltic Region History and Archaeology, Klaipėda University, Herkaus Manto Str. 84, LT-92294 Klaipeda, Lithuania; daugnora@gmail.com; 4 Department of Historical Sciences, Institute of Archaeology, Nicolaus Copernicus University, Szosa Bydgoska 44/48, 87-100 Torun, Poland; makdan@umk.pl

**Keywords:** raccoon dog, teeth measurements, tooth rows, sexual dimorphism

## Abstract

Sex identification is often challenging, especially when only skeletal remains are available. While previous research has primarily focused on skull measurements, dental traits have received less attention. This study aimed to measure the upper and lower canines, molars, and tooth rows of raccoon dogs in Lithuania to examine sexual dimorphism and analyze the correlation between different teeth and tooth row measurements. A total of 90 skulls with lower jaws of adult raccoon dogs were examined, including 55 males and 35 females. Osteometric analysis followed standard protocols, with canine measurements adapted using a method tailored to the dentition of carnivorous species. Of all the study measurements, the canine teeth demonstrated the most significant sex differences. The correlation analysis showed a strong correlation (*p* < 0.001) between the dimensions of the upper molars P^4^, M^1^, and M^2^. The upper tooth rows were strongly correlated (*p* < 0.001) with the dimensions of the P^4^ and canines. Lower molar correlations were weaker than upper ones, and lower tooth rows showed less correlation with tooth measurements. These findings indicate that molars and tooth rows are not reliable for sex determination in raccoon dogs. However, the observed sexual dimorphism in canine teeth may offer insights for future zooarchaeological and comparative anatomical studies.

## Simple Summary

The raccoon dog is one of the most widespread invasive members of the canine family. However, its teeth have received less attention in morphometric studies compared to the skull. This study aimed to measure canine and molar teeth, as well as tooth rows, to identify potential differences between males and females. Additionally, we analyzed the correlation between different teeth and tooth row measurements. The results showed that teeth differences between sexes were minimal, with the most significant variation found in canine teeth. Tooth row measurements did not differ between sexes. Furthermore, we observed that upper teeth and tooth rows had stronger correlations than lower ones.

## 1. Introduction

A raccoon dog is one of Lithuania’s most common representatives of the canine family [1]. It is an introduced species that first entered Lithuania in 1948 from Belarus and later from Latvia. By 1960, it had spread throughout the country [2]. Their sexual maturity occurs at 9–11 months of age [3,4]. Like other representatives of the canine family, raccoon dogs have a typical dental formula: I 3/3, C 1/1, P 4/4, M 2/3 [5]. A noticeably more frequent trend in raccoon dogs is that the lower M_3_ molar does not erupt. This dental anomaly was found to be the most common one, not only in raccoon dogs [6] but also in red foxes [7].

Raccoon dogs are omnivores and feed on small rodents and invertebrates. The diet of raccoon dogs living in Lithuania depends on the season; in the warm season, it consists of season-predominating plants and amphibians; in the cold season—ungulate carrion, plants in a smaller quantity, and rodents make up the bulk of raccoon dogs’ diet [8]. As other studies have shown, diet affects the development of raccoon dogs’ teeth, especially molars. Changes are observed if the diet contains a more considerable amount of solid food, e.g., insects with thick chitin [9].

The morphometric studies on raccoon dog teeth conducted in other countries focused on measurements of the skulls and mandibles, with less attention paid to the teeth. Several studies examined the skulls and teeth of the European raccoon dog subspecies (*N. p. ussuriensis*). A morphometric study of the skulls and teeth of raccoon dogs living in Finland was also performed. At the same time, the data were compared with the skull and teeth dimensions of another subspecies (*N. p. viverrinus*) [10]. Another study, which compared the measurements of the skulls and selected teeth of raccoon dogs living in various regions of Russia and Poland, also focused on skull measurements [11]. Another study conducted in Poland compared the skull dimensions of raccoon dogs to determine sex differences but did not measure teeth [12]. The same study examined the correlation between individual skull dimensions. More detailed studies on teeth measurements were also conducted but on different subspecies of raccoon dogs, *N. p. koreensis* and *N. p. albus* [8,13].

In Lithuania, a preliminary study with a small sample size (10 males and five females) provided initial insights into the skull and molar measurements of raccoon dogs [14]. Subsequently, Griciuvienė et al. (2013) [15] conducted a comparative morphometric analysis of 36 raccoon dog skulls from Lithuania but did not include dental measurements, relying instead on descriptive statistical methods. This highlights a clear scientific need to focus on the teeth of male and female raccoon dogs, as they are critical for carnivorous function. Despite their importance, canines have been largely overlooked in previous research. Furthermore, the increasing prevalence of unerupted M_3_ lower molars suggests that including this dimension could be valuable for future comparative studies and monitoring morphological changes over time. It should be emphasized that teeth are among the best-preserved bony structures in fossil material, making dental studies crucial in zooarchaeology [16]. The search for metric criteria of sexual dimorphism is particularly significant for subfossil collections of carnivorous mammals, where complete skulls, pelvises, or penis bones are often absent [17,18]. Our study of contemporary raccoon dog collections contributes to understanding morphological repeatability and dimorphism, aiding in the accurate sex categorization of subfossil remains. Focusing on key functional teeth—canines, carnassials, and molars—this research enhances carnivore identification. Future studies will expand the dataset with broader dental measurements and advanced morphometric methods.

In this context, the present study aimed to measure the upper and lower canines, molars, and tooth rows of raccoon dogs in Lithuania to examine sexual dimorphism and analyze the correlation between different teeth and tooth row measurements.

## 2. Material and Methods

The investigated skulls of raccoon dogs (*Nyctereutes procyonoides*) belong to the Kaunas Tadas Ivanauskas Museum of Zoology, Lithuania. The skull collection was started in 1955 and is constantly added with new skulls. The skulls are kept in the cabinets and organized in numbered drawers. Each skull has an attached registration card with basic data (collection site, body length, weight, and sex). A male skull usually has the penis bone (os penis) attached to it. The skulls were obtained from hunters or found during expeditions.

A total of 90 skulls and mandibles were investigated (*n* = 90): 55 males (*n* = 55) and 35 females (*n* = 35). In this study, we examined only the skulls of adult raccoon dogs. The maturity of the raccoon dog skulls was determined by the closure of the presphenoid-vomer and basisphenoid-presphenoid sutures [19]. In carnivorous mammals, permanent teeth after eruption remain the same size throughout their whole life (except for fractures or wear), so the raccoon dog samples were not divided according to age [20]. In this study, only well-preserved teeth with no noticeable wear were included. Since crown height was not measured, slight wear was allowed. We selected skulls with fully erupted lower third molars.

**Figure 1:**
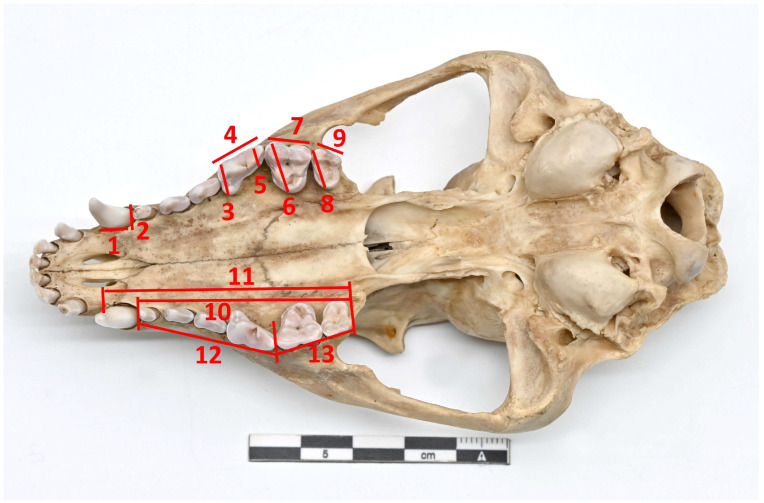
Method of measurement of upper canine, molar teeth, and upper tooth rows. C. 1. Length (UC_L); 2. Width (UC_B). P^4^. 3. Maximum width (UP4_GB); 4. Length (UP4_L); 5. Minimum width (UP4_B). M^1^. 6. Width (UM1_B); 7. Length (UM1_L). M^2^. 8. Width (UM2_B); 9. Length (UM2_L). 10. UDR–1: length of the upper cheek tooth row (rostral border of alveolus of P^1^—caudal border of alveolus of M^2^). 11. UDR–2: rostral border of alveolus of C—caudal border of alveolus M^2^. 12. U–PRE: length of the upper premolar row (rostral border of alveolus of P^1^—caudal border of alveolus P^4^). 13. U–MOL: length of the upper molar row (rostral border of alveolus of M^1^—caudal border of alveolus M^2^).

**Figure 2:**
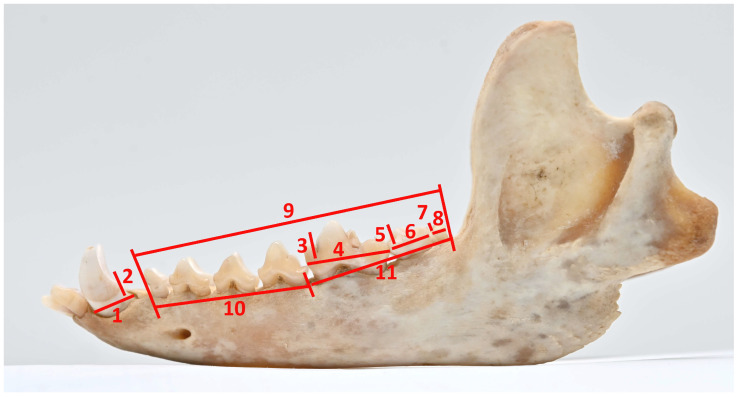
Method of measurement of lower canine, molar teeth, and lower tooth rows. C. 1. Length (LC_L); 2. Width (LC_B). M_1_. 3. Width (LM1_B); 4. Length (LM1_L). M_2_. 5. Width (LM2_B); 6. Length (LM2_L). M_3_. 7. Width (LM3_B); 8. Length (LM3_L). 9. LDR: length of the lower cheek tooth row (rostral border of alveolus of P_1_—caudal border of alveolus of M_3_). 10. L–PRE: length of the lower premolar row (rostral border of alveolus of P_1_—caudal border of alveolus P_4_). 11. L–MOL: length of the lower molar row (rostral border of alveolus of M_1_—caudal border of alveolus M_2_).

The osteometric analysis was performed using the A. von den Driesch (1976) [21] method, except for canine teeth, which were measured according to the methodology taken by Szuma (2000) [22]. Measurements were taken using a digital caliper with an accuracy of 0.01 mm, focusing on the left side of the upper and lower teeth and tooth rows. The canines and molars, including carnassials (*dentes sectorii*) (upper—C, P^4^, M^1^, M^2^ and lower—C, M_1_, M_2_, M_3_), were selected for the study measurements because they are essential in carnivorous diets [23]. Furthermore, M_1_ is a common research object in paleontological studies [24]. The measurements of individual teeth and dental rows are shown in Figure 1 and Figure 2.

Statistical analysis. The data analysis was performed using SPSS 24.0. The Kolmogorov–Smirnov test (with Lilliefors Significance Correction) set the normal distribution of the analyzed data. Average and standard deviation were evaluated as the main parameters of descriptive analysis. The average differences between the female and male groups were expressed in percentages. The statistical validity of the data was evaluated using Student’s *t*-test.

We performed the discriminant analysis (DA) and principal component analysis (PCA) to detect the presence or absence of any correlation between the raccoon dogs’ sex and their teeth sizes. Pearson’s correlation coefficient was calculated using the combined data from male and female subjects to examine the correlation between teeth and teeth row measurements. The data were regarded as statistically significant when *p* < 0.05.

## 3. Results

Data normality. The Kolmogorov–Smirnov test revealed that the data followed a normal distribution.

Descriptive statistics. The means, standard deviations, and reliability of the measurements of male and female teeth and tooth rows are presented in Table 1. The analysis revealed minor differences between male and female measurements. The length of the upper canines, UC_L (3.4% *p* < 0.05), and width, UC_B (4.9% *p* < 0.01), and the width of the lower canines, LC_L (4.1% *p* < 0.05), were larger in males than in females. Meanwhile, the parameters of the lower molar M_3_, LM3_L (11.1% *p* < 0.01), and LM3_B (8.1% *p* < 0.01) were larger in females than in males. The difference in the dimension of only one tooth row was statistically significant; the length of the upper premolar row, U_PRE (3.2% *p* < 0.05), was larger in males than females.

Discriminant analysis (DA). The Wilks’ Lambda of individual measurements ranged from 0.882 to 1, which indicated that, based on the available data, it was difficult to classify the objects by sex reliably. The LM3_L and LM3_B values would allow for the best data classification (Wilks’ Lambda 0.886 and 0.882, respectively; *p* < 0.01). We could distinguish statistically significantly by UC_L (Lambda 0.952; *p* < 0.05) and UC_B (Lambda 0.930; *p* < 0.01). The discriminant function for other features was minimal, with values approaching 1, as shown in Table 2.

The enter independents together DA (Table 3), which included all tooth measurements, yielded the best result—the lowest Wilks’ Lambda value of 0.574 (*p* < 0.005) and the highest canonical correlation value of 0.652, which showed that such a model best differentiated our studied objects. The model correctly classified 82.9% of the study objects—73.5% of the female and 89.6% of the male group objects. However, the Box’s *M* Tests showed that the covariance matrices of the compared groups were unequal (F = 1.156; *p* < 0.05), so we cannot fully rely on the discriminant function results.

The enter stepwise together DA using the model Box’s *M* Tests showed that the covariance matrices of the compared groups did not differ (F = 1.405; *p* = 0.208); see Table 3. During the analysis, three main measurement values were identified, according to which we could classify raccoon dogs into females and males. The Wilks’ Lambda of the model was 0.882 (*p* < 0.01) after including one parameter LM3_B, it decreased to 0.824 (*p* < 0.001) after additionally including LC_L and reached 0.774 (*p* < 0.001) after adding U_MOL to the model. The inclusion of other measurements was insignificant. The model correctly classified 71.1% of the study objects, 57.1% of the female and 80.0% of the male group objects. Leaving only the three essential measurements (LM3_B, LC_L, and U_MOL) in the model significantly reduces the discriminant function (variance of 0.2).

Principal component analysis (PCA). The PCA identified six principal components that explained 73.7% of the total variance in the data. The first component explained 22.0%, the second 14.4%, the third 3.8%, the fourth 9.8%, the fifth 7.3%, and the sixth 6.6%.

Table 4 presents the data grouped by their component membership, with the first component containing the most features (9), followed by the second (5), the third (4), and the fourth, fifth, and sixth components, each comprising two data points. We observed that there was no obvious grouping of measurements into components, neither according to the upper or lower jaw nor according to the measurements in the same anatomical region.

**Figure 3:**
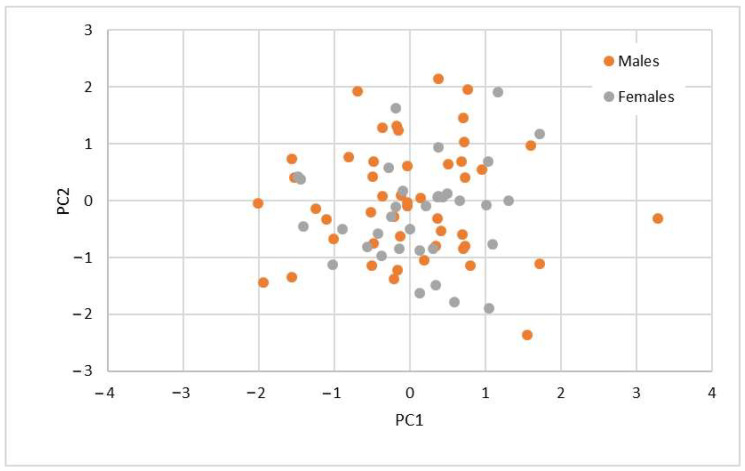
PC1 and PC2 dispersion and comparison between females and males.

In Figure 3, neither the first nor the second principal component clearly separates males from females; the data overlap. Consequently, the PCA does not facilitate the effective grouping of data points into descriptive categories nor does it distinguish between males and females.

**Correlation analysis.** From the correlation data of upper teeth and tooth rows (Table 5), we observed a strong correlation (*p* < 0.001) between the parameters of the P^4^, M^1^, and M^2^ molars. In contrast, the parameters of the canines strongly correlated (*p* < 0.001) with the parameters of the P^4^ and M^1^ molars but did not correlate with the parameters of the M^2^. The upper tooth rows (UDR-1 and UDR-2) strongly correlated (*p* < 0.001) with the parameters of the P^4^ and canines. The molar row (U_MOL) strongly correlated (*p* < 0.001) with all tooth parameters, except for the width of the canine, with which the correlation was weaker (*p* < 0.05).

The correlation data between the measurement results of the lower teeth and tooth rows are presented in Table 6. The correlation between the molar teeth measurements was weaker than that of the upper teeth; the lower molar M_3_ correlated strongly (*p* < 0.001) only with the molar M_2_; the correlation with M_1_ was very weak (LM1_L) or negative (LM1_B). The data on the canines correlated most with M_1_, while the correlation with M_3_ was very weak. The measurements of the lower tooth rows correlated less weakly with the teeth than with the upper tooth rows. The correlation of the length of the premolar row (L-PRE) was weak or negative; the length of the molar row correlated with the measurements of the teeth statistically significantly. However, a strong correlation was much less common than in the case of the length of the upper molar tooth row.

## 4. Discussion

In many carnivorous species, such as wolves, wild cats, and badgers, sexual dimorphism in skull size is well pronounced, with male skulls typically larger than those of females [16,23,25]. However, it was found that monogamous predator species (raccoon dogs are strictly monogamous) featured low sexual dimorphism [26]. We also confirmed this in our previous study, in which the measurements of the raccoon dog skulls were compared, out of 27 measurements, only 2 were statistically significant [14]. Although we investigated and measured a significantly larger quantity of skulls in this study than in our previous one (15 vs. 90), the obtained results on the tooth rows differed slightly between the raccoon dog sexes. Despite the statistically significant difference in one measurement (U-PRE), the DA and PCA showed that the differences in the tooth rows were not valid for identifying sex. Griciuvienė et al. (2013) [15] measured and compared the dimensions of the skulls of Lithuanian raccoon dogs from the 1957 collection and found that the lower tooth row of males was longer than that of females (*p* < 0.04). However, in this study, with the more extensive collection of skulls collected over a more extended period (from 1955 to 2022), we did not find any statistically significant differences in the measurements of the lower tooth row. Kim SangIn et al. (2012) [13] obtained similar results comparing the measurements of the skulls of *N. p. koreensis* between sexes. Only two measurements, the postorbital constriction and lower jaw thickness of the skull, were statistically significant. The differences in the measurements of the tooth rows were statistically insignificant. In conclusion, we can state that skull measurements are unsuitable for sex identification.

After analyzing the measurements of the teeth examined in this study, we found statistically significant differences between the dimensions of the canines—the upper canines of males were longer and broader, and the lower ones were longer than those of females. Meanwhile, the dimensions of the lower M_3_ molar, on the contrary, were larger in females than in males. Similar results were obtained in the study, which measured the skulls and teeth of another subspecies of raccoon dog, *N. p. koreensis*. The study authors emphasized that the most indicative dimorphic measurements were the widths of the upper and lower canines, which were around 8% larger in male specimens on average. Similarly to our study, the length of the lower M_3_ molar was greater in females than in males [13]. A comprehensive study of the sexual and territorial differences in the skull and teeth of raccoon dogs from Poland, and various regions of Russia showed that the greatest sexual dimorphism was found comparing the teeth, not skull, dimensions. Although this study measured only the length of the upper canines and M^1^ molar, it was essential to confirm that the length of the upper canines was greater in males than in females. This measurement was characterized by the highest index of sexual size dimorphism [11]. Also, our study found statistically significant differences in the measurements of the canine tooth. Considering the fact that even in other subspecies of raccoon dogs, the canine tooth measurements showed the most prominent sexual dimorphism, it can be stated that the greatest differences between the sexes are pronounced in the canine teeth. Although statistically significant differences were found in the dimensions of the M_3_ lower molar, the discriminant and principal component analyses showed that this tooth was unsuitable for gender identification. The studies of other canid families confirmed that the M_3_ lower molar in red foxes was the most variable tooth, while sex dimorphism in the canine teeth area was higher than in the carnassial one [22]. Sexual dimorphism is also observed in other carnivores. Hatlauf et al. (2021) [27] reported significant sexual size dimorphism in golden jackals (*Canis aureus*) and African wolves (*Canis lupaster*). Their study found that the mesiodistal diameter of the upper canine was the most effective trait for distinguishing sexes in these species. We considered including another measurement of canine teeth, i.e., height, because Gittleman and Van Valkenburgh, (1997) [28] indicated the possibility that male canine teeth might be much larger in one dimension, namely the height of the canine tooth crown. However, we abandoned this examination after taking preliminary measurements because the canine teeth’s apex was usually deformed (chipped and/or worn), which could result in inaccuracies.

The correlation data analysis showed that there was a strong correlation (*p* < 0.001) between the measurements of the molars in the carnassial-molar area (P^4^–M^2^). Meanwhile, the correlation between the measurements in the lower molar area (M_1_–M_3_) was weaker than that of the upper teeth. Other authors have not sufficiently studied the correlation pattern of teeth in raccoon dogs, so we refer to the studies of representatives of different canine species. Unlike raccoon dogs, Gingerich and Winkler, (1979) [29] found that the most highly correlated tooth pairs are in the premolar series, where no tooth-to-tooth occlusion is possible in red foxes. The most highly correlated teeth in the lower dentition of red foxes are P_2_–P_3_–P_4_, and in the upper dentition, P^2^–P^3^. Szuma, (2000) [22] also found the strongest between-tooth correlations in the premolar area. Since our study did not examine premolars, we cannot confirm whether a similar pattern is also a characteristic of raccoon dogs.

## 5. Conclusions

In summary, we can state that there are no sex differences in the molars, especially the tooth rows, of raccoon dogs. Of all our measurements, the canine teeth indicated the greatest sex differences, but based on the DA and PCA analyses, these teeth were also unsuitable for sex identification. However, having the collected material of suitable canines for crown height measurement would allow for extended studies in the future, including the teeth of the premolar (P1–P4) group. Expanding the skull sample size would further enhance these studies, providing a broader dataset for more comprehensive analysis. Additionally, future research on raccoon dogs should include geometric morphometrics, as linear morphometric methods have shown limited effectiveness in detecting sexual dimorphism.

## Figures and Tables

**Table 1:** Difference between the mean of teeth measurements in female and male raccoon dogs.

	Males; *n* = 55 Mean (Std. Dev.)	Females; *n* = 35 Mean (Std. Dev.)	Mean Difference	*p*-Value (Student *t*-Test)
UP4_L	10.58 (0.6)	10.33 (0.67)	2.5%	0.061
UP4_GB	5.36 (0.54)	5.46 (0.41)	−1.8%	0.355
UP4_B	4.46 (0.34)	4.43 (0.26)	0.7%	0.668
UM1_L	7.94 (0.66)	7.97 (0.52)	−0.4%	0.817
UM1_B	8.85 (0.56)	8.79 (0.49)	0.7%	0.618
UM2_L	4.37 (0.40)	4.56 (0.57)	−4.2%	0.090
UM2_B	6.26 (0.50)	6.40 (0.63)	−2.1%	0.258
UC_L	5.67 (0.42)	5.48 (0.39)	3.4%	*p* < 0.05
UC_B	4.10 (0.38)	3.91 (0.26)	4.9%	*p* < 0.01
UDR-1	38.39 (1.38)	38.08 (0.98)	0.8%	0.256
UDR-2	45.47 (1.46)	44.99 (1.17)	1.1%	0.107
U-PRE	27.12 (2.55)	26.28 (0.83)	3.2%	*p* < 0.05
U-MOL	13.15 (0.75)	13.4 (0.72)	−1.8%	0.125
LM1_L	12.46 (0.54)	12.37 (0.47)	0.7%	0.430
LM1_B	5.00 (0.31)	4.98 (0.30)	0.5%	0.733
LM2_L	6.14 (0.43)	6.30 (0.44)	−2.5%	0.094
LM2_B	4.05 (0.24)	4.08 (0.36)	−0.7%	0.666
LM3_L	2.57 (0.47)	2.89 (0.58)	−11.1%	*p* < 0.01
LM3_B	2.19 (0.27)	2.38 (0.33)	−8.1%	*p* < 0.01
LC_L	5.75 (0.60)	5.52 (0.45)	4.1%	*p* < 0.05
LC_B	4.15 (0.45)	4.07 (0.37)	1.9%	0.383
LDR	44.51 (1.53)	44.32 (1.34)	0.4%	0.539
L-PRE	22.99 (1.14)	22.75 (0.78)	1.0%	0.243
L-MOL	21.40 (1.01)	21.38 (1.28)	0.1%	0.925

**Table 2:** Discriminant analysis: canonical discriminant function and Wilks’ Lambda evaluation.

	Canonical Discriminant Function Unstandardized Coefficients	Wilks’ Lambda	*p*-Value
UP4_L	−0.199	0.951	0.061
UP4_GB	1.731	0.988	0.355
UP4_B	−0.247	0.998	0.668
UM1_L	0.465	0.999	0.817
UM1_B	−0.729	0.999	0.618
UM2_L	0.389	0.963	0.067
UM2_B	0.153	0.981	0.258
UC_L	−0.396	0.952	*p* < 0.05
UC_B	−1.199	0.930	*p* < 0.01
UDR-1	−0.699	1.000	0.256
UDR-2	0.289	0.984	0.107
U-PRE	0.833	0.984	0.065
U-MOL	−0.820	0.964	0.125
LM1_L	0.239	0.989	0.430
LM1_B	0.370	0.993	0.733
LM2_L	−0.817	0.972	0.094
LM2_B	0.507	0.996	0.636
LM3_L	2.012	0.886	*p* < 0.01
LM3_B	−1.342	0.882	*p* < 0.01
LC_L	1.516	0.962	0.058
LC_B	0.093	0.998	0.383
LDR	−0.259	1.000	0.539
L-PRE	−0.306	0.994	0.282
L-MOL	0.389	1.000	0.925

**Table 3:** Discriminant analysis, the model with Box’s *M.*

DA Model and Analyzed Measurements	Wilks’ Lambda	Canonical Correlation	Correct Classification of Original Cases (%)			Box’s M
			Total	Female	Male	
Enter independents together (all measurements)	0.574 (*p* < 0.05)	0.652	82.9%	73.5%	89.6%	F = 1.156 *p* < 0.05
Enter independents together (only upper teeth and rows)	0.743 (*p* < 0.05)	0.532	75.9%	61.8%	85.7%	F = 1.267 *p* = 0.056
Enter independents together (only lower teeth and rows)	0.807 (*p* = 0.089)	0.439	73.3%	65.7%	78.2%	F = 1.325 *p* < 0.05
Enter stepwise together	0.774 (*p* < 0.001)	0.475	71.1%	57.1%	80.0%	F = 1.405 *p* = 0.208

**Table 4:** Principal component analysis.

	PC1	PC2	PC3	PC4	PC5	PC6
UP4_GB	0.817	0.235	0.077	−0.021	0.022	−0.175
U-MOL	0.761	−0.069	0.211	−0.069	−0.038	0.158
UM2_B	0.687	−0.143	−0.094	0.305	0.033	−0.043
UM1_B	0.673	0.126	0.317	0.230	0.139	0.100
LM2_B	0.666	−0.099	0.189	0.379	0.326	0.086
LM1_L	0.652	0.213	0.242	0.365	0.032	0.298
UM1_L	0.634	0.027	0.382	0.157	0.101	0.186
LM1_B	0.625	0.039	0.207	0.200	−0.017	0.221
UP4_L	0.581	0.262	0.406	0.300	−0.024	0.072
U-PRE	−0.259	0.850	0.004	0.213	−0.039	−0.044
L-PRE	0.042	0.839	−0.136	−0.138	0.122	−0.174
UDR-1	0.274	0.833	0.054	0.002	−0.006	0.178
LDR	0.164	0.707	−0.100	0.461	0.040	0.109
UDR-2	0.033	0.692	0.468	−0.139	−0.210	0.281
LC_B	−0.020	−0.007	0.802	0.305	0.217	−0.046
UC_B	0.270	−0.078	0.785	−0.069	−0.042	0.052
LC_L	0.426	0.001	0.748	0.096	0.188	0.174
UC_L	0.332	0.055	0.722	−0.139	−0.270	0.001
LM2_L	0.279	0.086	0.123	0.784	−0.051	0.019
L-MOL	0.308	0.047	−0.027	0.783	0.259	0.205
LM3_L	−0.033	−0.034	−0.022	−0.007	0.883	0.317
UP4_B	0.462	0.065	0.139	0.255	0.636	−0.200
LM3_B	0.074	0.260	−0.023	0.106	0.359	0.775
UM2_L	0.418	−0.179	0.268	0.187	−0.064	0.593
Eigenvalues	33.5	13.9	10.2	6.7	5.0	4.5
Rotation sums of squared loadings % of variance	22.0	14.4	13.8	9.8	7.3	6.6
Rotation sums of squared loadings cumulative %	22.0	36.3	50.1	59.9	67.2	73.7

**Table 5:** Correlation between upper teeth and tooth row measurements.

	UP4_GB	UP4_B	UM1_L	UM1_B	UM2_L	UM2_B	UC_L	UC_B	UDR-1	UDR-2	U-PRE	U-MOL
UP4_L	0.435 ***	0.304 **	0.420 ***	0.450 ***	0.307 **	0.282 **	0.463 ***	0.511 ***	0.322 **	0.464 ***	0.239 *	0.332 ***
UP4_GB		0.380 ***	0.391 ***	0.509 ***	0.251 *	0.490 ***	0.367 ***	0.375 ***	0.330 **	0.286 **	0.188	0.408 ***
UP4_B			0.368 ***	0.447 ***	0.198	0.352 ***	0.157	0.177	0.161	0.100	0.074	0.333 ***
UM1_L				0.568 ***	0.344 ***	0.415 ***	0.409 ***	0.382 ***	0.324 **	0.261 *	0.071	0.667 ***
UM1_B					0.425 ***	0.591 ***	0.318 **	0.444 ***	0.318 **	0.312 **	0.162	0.460 ***
UM2_L						0.465 ***	0.179	0.127	0.218 *	0.210 *	0.116	0.405 ***
UM2_B							0.154	0.206	0.258 *	0.180	0.152	0.402 ***
UC_L								0.645 ***	0.309 **	0.534 ***	0.190	0.357 ***
UC_B									0.292 **	0.475 ***	0.172	0.228 *
UDR-1										0.823 ***	0.568 ***	0.313 **
UDR-2											0.494 ***	0.245 *
U-PRE												0.090

*—*p* < 0.05; **—*p* < 0.01; ***—*p* < 0.001.

**Table 6:** Correlation between lower teeth and tooth row measurements.

	LM1_B	LM2_L	LM2_B	LM3_L	LM3_B	LC_L	LC_B	LDR	L-PRE	L-MOL
LM1_L	0.551 ***	0.431 ***	0.432 ***	0.002	0.169	0.536 ***	0.316 **	0.349 ***	0.142	0.385 ***
LM1_B		0.172	0.365 ***	−0.113	−0.034	0.442 ***	0.240 *	0.166	0.067	0.205
LM2_L			0.480 ***	0.277 **	0.397 ***	0.169	0.191	0.270 *	−0.073	0.429 ***
LM2_B				0.249 *	0.344 ***	0.312 **	0.266 *	0.319 **	0.051	0.474 ***
LM3_L					0.717 ***	0.024	0.108	0.097	−0.106	0.244 *
LM3_B						0.113	0.120	0.260 *	−0.061	0.344 ***
LC_L							0.629 ***	0.301 **	0.150	0.242 *
LC_B								0.278 **	0.081	0.276 **
LDR									0.571 ***	0.703 ***
L-PRE										0.082

*—*p* < 0.05; **—*p* < 0.01; ***—*p* < 0.001.

## Data Availability

The authors confirm that all relevant data included in the article are available from the corresponding author upon a reasonable request.
